# Molecular mechanism by which *Apis cerana cerana* MKK6 (*AccMKK6*)-mediated MAPK cascades regulate the oxidative stress response

**DOI:** 10.1042/BSR20181301

**Published:** 2018-12-11

**Authors:** Xinxin Wang, Chen Wang, Xuepei Cui, Lijun Wang, Zhenguo Liu, Baohua Xu, Han Li

**Affiliations:** 1 State Key Laboratory of Crop Biology, College of Life Sciences, Shandong Agricultural University, Taian, Shandong 271018, P.R. China; 2 College of Animal Science and Technology, Shandong Agricultural University, Taian, Shandong 271018, P.R. China

**Keywords:** Apis cerana cerana, AccMKK6, Oxidative resistance, RNA interference, Yeast two-hybrid system

## Abstract

Mitogen-activated protein kinase kinases (MKKs) are important components of the MAPK signaling pathways, which play a key role in responding to stress and inflammatory stimuli. Here, a new MKK gene, *AccMKK6*, was identified and functionally analyzed in *Apis cerana cerana*. Real-time quantitative PCR (qPCR) and Western blot analysis demonstrated that the AccMKK6 expression level was up-regulated by several environmental stresses. Moreover, the knockdown of *AccMKK6* by RNA interference technology altered the expression levels of some antioxidant genes. In addition, the knockdown of *AccMKK6* resulted in increased malonyldialdehyde (MDA) concentration and decreased antioxidant-related enzymes activity in honeybees. To explore the MAPK signaling pathways involved in *AccMKK6*, we identified the transcription factor kayak in *A. cerana cerana*. We analyzed the interactions of AccMKK6, Accp38b, and Acckayak using the yeast two-hybrid system. *AccMKK6* and *Acckayak* showed similar expression profiles after several stress treatments. In addition, the expression level of *Acckayak* was significantly increased when *AccMKK6* was silenced. Therefore, we speculate that *AccMKK6* may be involved in the MAPK cascades, which play a crucial role in counteracting oxidative stress caused by external stimuli.

## Introduction

Insects face a variety of adverse environmental pressures, including global warming, heavy metal pollution and the excessive use of pesticides. These environmental stresses can enhance the generation of reactive oxygen species (ROS), which cause oxidative damage [1–3]. ROS are formed as byproducts of aerobic metabolism, including superoxide anions, hydrogen peroxide, and hydroxyl radicals [4]. Generally, ROS are the second messenger in intracellular signaling pathways and maintain a dynamic equilibrium under normal conditions [5]. However, this equilibrium can be disrupted by adverse environmental stresses, which result in lipid peroxidation and in protein and DNA damage and can adversely affect cell viability by causing membrane damage and enzyme inactivation [6]. To maintain ROS homeostasis and prevent oxidative damage, organisms have developed complex antioxidant mechanisms to avoid oxidative damage [7]. Mitogen-activated protein kinase (MAPK) signaling pathways are critical in responding to oxidative stress [8,9].

MAPK signaling pathways transfer extracellular information to the interior of the cell, regulating cell activities in response to external stimuli [10,11]. These signaling pathways are activated by a variety of stimuli including cytokines, hormones, and diverse cellular stressors such as oxidative stress and endoplasmic reticulum (ER) stress [12]. MAPK signaling pathways have been implicated in cancer, neurodegenerative diseases, and the pathogenesis of several chronic diseases, as the accumulation of ROS and the activation of MAPK signaling pathways are considered to lead to disease in humans via the induction of apoptosis by various mechanisms [13]. Classical MAPK signaling pathways have been identified in mammalian cells, including the ERK, JNK, and p38 MAP kinase pathways [14,15]. Each MAPK signaling pathway consists of at least three components, MAPK kinase kinases (MKKKs), MAPK kinases (MKKs), and MAPKs. MKKKs phosphorylate and activate MKKs, which in turn phosphorylate and activate MAPKs [13]. Thus, MKKs can be considered ‘signaling hubs’ that regulate the MAPK cascades.

MKKs are serine/threonine protein kinases that are expressed in various tissues and cells of eukaryotes [16]. MKKs are activated by MKKKs via dual phosphorylation sites on Ser and Thr [17]. There are only four MKKs in *Drosophila*, namely, Dsor1 (MKK1/2), Lic (MKK3/6), Sek (MKK4), and Hep (MKK7) [18]. MKK1/2 function in defense signaling, mediated by MKKK1, MAPK4, and MKS1 [19]. MKK7 has also been found to trigger inflammation, oxidative stress, and apoptosis responses by directly activating the expression of JNK and mediating MKK7/JNK signaling [20]. The cascade comprising the protein kinases ASK1, MKK4, and JNK induces cell death in response to oxidative stress [21]. Previous studies have reported that MKK6 plays an important role in inflammation and oxidative stress responses. Recently, the gene *AjMKK3/6* was isolated from *Apostichopus japonicus* and induced by *V. splendidus* as an immune response [22]. MKK6 can participate in the response to a wide variety of environmental and biological stresses, including ROS, ER stress, and calcium overload, upon activation by ASK1 [23]. There are few studies on MKK6 in insects, and none have been reported on the function of MKK6 in *Apis cerana cerana*. Therefore, understanding the role of MKK6 in resisting external pressure could provide a basis for studying the antioxidant system of *A. cerana cerana*.

*A. cerana cerana* is the major honeybee species in China and plays an important role in ecological balance and the agricultural industry. *A. cerana cerana* has the advantages of a long period of collecting honey, low food cost and good resistance to disease [24,25]. However, in recent years, the survival of *A. cerana cerana* has been seriously threatened due to various environmental stresses including the indiscriminate use of pesticides, infectious diseases, and global warming [26]. These adverse environmental stresses can lead to oxidative damage in honeybees. Understanding the antioxidant system and its defense mechanism is of great significance in the development of the *A. cerana cerana* population. Studies have found that MKK6 may play a role in protecting organisms from ROS [23]. Accordingly, we isolated *AccMKK6* from *A. cerana cerana*. To further elucidate the function of *AccMKK6* in oxidative stress, we performed gene expression analyses, and RNA interference (RNAi) experiments and analyzed the concentration of malonyldialdehyde (MDA) and the activities of superoxide dismutase (SOD) and peroxidase (POD) *in vivo* after *AccMKK6* knockdown. Due to the peculiar relationship between MKK6, p38 and the downstream targets of p38, we cloned the transcription factor kayak and proved the interactions between AccMKK6, Accp38b, and Acckayak. *Acckayak* expression was increased after *AccMKK6* silencing. Based on our results, we concluded that *AccMKK6* is involved in the MAPK cascades and plays a protective role in oxidative stress.

## Materials and methods

### Insects and treatments

The honeybees (*A. cerana cerana*) used in this study were obtained from the experimental apiary of the College of Animal Science and Technology, Shandong Agricultural University (Taian, Shandong, China). Larvae [first (L1) and fourth (L4) instar larvae], pupae [prepupae (PP), white-eyed (Pw), pink-eyed (Pp), brown-eyed (Pb), and dark-eyed (Pd) pupae] and adult workers were collected. The experimental honeybees were kept in bee boxes at a constant temperature of 32°C and 80% humidity under a 24 h dark regimen [27]. The adult workers were divided into nine groups of 48 individuals each. Each group was exposed to different harmful conditions. Group 1 was injected with 1 μl of 20 mM H_2_O_2_ solution and analyzed after 0, 0.5, 1, 1.5, 2, or 2.5 h. The honeybees in groups 2 and 3 were injected with 1 μl of HgCl_2_ and CdCl_2_ (3 mg/ml), respectively, and analyzed after 0, 0.5, 1, 1.5, 2, or 2.5 h. Groups 4–6 of honeybees were injected with 1 μl of pesticide (paraquat, emamectin benzoate, acetamiprid) at concentrations of 20 μl/ml. Treated at 0 h honeybees were used as controls. The treated materials were immediately snap-frozen in liquid nitrogen and stored at −70°C.

### RNA extraction and cDNA synthesis

Total RNA was extracted from the honeybees using RNAiso Plus (TaKaRa, Japan) and then stored at −70°C. The RNA was reverse transcribed into first-strand complementary DNA (cDNA) using 5× All-In-One RT MasterMix with the AccuRT Genomic DNA Removal Kit (Applied Biological Materials Inc., Richmond, BC, Canada), which facilitates the elimination of contaminating genomic DNA from RNA.

### Isolation of the *AccMKK6* and *Acckayak* open reading frame sequences

To clone the open reading frame (ORF) sequences of *AccMKK6* (GenBank accession no. XP_016921098.1) and *Acckayak* (GenBank accession no. XP_016911846.1), specific primers (as shown in Supplementary Table S1) were designed based on the *AccMKK6* and *Acckayak* gene sequences and synthesized by Biosune Biotechnological Company (Shanghai, China). The PCR mixtures were as described by Zhao et al. [28], and the amplification conditions are shown in Supplementary Table S2. The PCR products were purified using a Gel Extraction Kit (Solarbio, Beijing, China), ligated with pEASY-T1 vectors (TransGen, Biotech, Beijing, China), and then transformed into *Escherichia coli* cells (DH5α) for sequencing. The primers used in this study are listed in Supplementary Table S1.

### Bioinformatics and phylogenetic analysis of *AccMKK6* and *Acckayak*

The amino acid sequences of *AccMKK6* and *Acckayak* were obtained from GenBank (https://www.ncbi.nlm.nih.gov/genbank/), and the isoelectric point and molecular mass were predicted by ExPASy (https://www.expasy.org/). Homologous *AccMKK6* and *Acckayak* amino acid sequences from other species were obtained using the BLAST search from the NCBI (https://blast.ncbi.nlm.nih.gov/Blast.cgi), and multiple amino acid sequence alignments were performed using DNAMAN version 6.0.3. A neighbor-joining (NJ) phylogenetic tree analysis was performed on the homologous sequences of MKKs from other species using MEGA version 4.1.

### Quantitative real-time PCR

To quantify the gene expression, real-time quantitative PCR was performed via the Bestar® One-step RT qPCR Kit (SyBR Green) (DBI Bioscience, China) and a CFX96TM Real-time PCR Detection System (Bio-Rad, Hercules, CA, U.S.A.). The specific qPCR primers for *AccMKK6* and *Acckayak* were designed based on the cDNA sequence. The housekeeping gene β-actin (GenBank accession no. HM_640276) was selected as an internal control [29]. The qPCR was performed in a total volume of 20 μl with 8 μl of double-distilled water, 1 μl of cDNA template, 0.5 μl of each primer, and 10 μl of Bestar® qPCR Mastermix. The qPCR program was as follows: 95°C for 30 s; 40 cycles of 95°C for 5 s, 55°C for 15 s, and 72°C for 15 s; and a final melt cycle from 55 to 96°C. All experimental samples were evaluated in triplicate, and the data were analyzed using CFX Manager Software version 1.1. The significant differences between the samples were identified by SPSS software version 17.0.

### The protein expression of *AccMKK6* and antibody preparation

Primers (M2F, M2R) containing *BamH*I and *Sac*I restriction enzyme sites were used to insert the ORF of *AccMKK6* into the expression vector pET-30a(+) (Novagen, Darmstadt, Germany). The recombinant plasmid pET-30a(+)-AccMKK6 was transformed into *E. coli* Transetta (DE3) (TransGen Biotech, Beijing, China). The bacterial solution was cultured in Luria Bertani (LB) with 50 μg/ml kanamycin at 37°C for 1–2 h until the cell density reached 0.4–0.6 at 600 nm. The expression of the recombinant AccMKK6 protein was induced with 2 mM isopropyl-1-thio-β-d-galactopyranoside (IPTG) at 28°C for 6-8 h, and the protein was then separated by 12% sodium dodecyl sulfate polyacrylamide gel electrophoresis (SDS-PAGE). To prepare an antibody against AccMKK6, the recombinant AccMKK6 protein band was excised from the gel, milled with 1 ml of normal saline and injected into white mice. The mice were injected once a week for a total of four injections. Blood was collected on the fourth day after the last injection and stored at 37°C for 1 h and then 4°C for 6 h. After centrifugation at 3000 ***g*** for 15 min, the supernatant antibodies were stored at −70°C.

### Protein extraction and Western blot analysis

The total protein was extracted from the honeybee samples using a Tissue Protein Extraction Kit (CWbiotech, Beijing, China). Western blotting was performed according to the procedure described by Li et al. [30] with some modifications. The protein samples were separated by 12% SDS-PAGE and electrotransferred onto a polyvinylidene difluoride (PVDF) membrane. The membrane was incubated overnight with the primary antibody (anti-AccMKK6) at 1:1000 (v/v) dilution. Tubulin was used as the reference antibody at a 1:5000 (v/v). The results were observed using the SuperSignal West Femto Trial Kit (Thermo Scientific, U.S.A.). The Western blot results were analyzed using ImageJ 1.51j8 (National Institutes of Health, U.S.A.).

### RNAi of *AccMKK6*

We used an RNAi experiment to knock down *AccMKK6* in adult workers. Primers were designed to select regions of lower homology, and 23 bp of the T7 promoter sequence (TAATACGACTCACTATAGGGCGA) was added to the start of the *AccMKK6* forward primer (M3F) and the end of the AccMKK6 reverse primer (M3R). The double-stranded RNAs (dsRNA) were prepared using the T7 RiboMAX^TM^ Express RNAi System (Promega, U.S.A.) according to the manufacturer’s instructions. We selected the green fluorescent protein gene (GFP, GenBank accession no. U87974), which has no homolog in the honeybees, as a control [31]. The dsRNA (6 μg/individual) was injected into adult honeybees between the first and second abdominal segments using a microsyringe [32]. In addition, no injected honeybees and honeybees injected with 1 μl of water were used as control groups. The silencing efficiency was determined by qPCR and Western blot.

### Transcription level analysis of antioxidant genes and enzymatic activities after *AccMKK6* knockdown

qPCR was performed to analyze the expression profiles of *AccSOD1* (GenBank ID: JN700517), *AccSOD2* (GenBank ID: JN637476), *AccGSTS4* (GenBank ID: JN008721), *AccCYP4G11* (GenBank ID: kc243984), *AccGSTO2* (GenBank ID: JX434029), and *AccGSTD* (GenBank ID: JF798573) after *AccMKK6* was knocked down. The total protein was extracted from the whole adult bees 36 h after they had been injected with dsRNA and quantified using the BCA Protein Assay Kit (Nanjing Jiancheng Bioengineering Institute, Nanjing, China). The MDA concentration was measured using an MDA Assay Kit (Nanjing JianCheng Bioengineering Institute, Nanjing, China). The activity levels of the enzymes SOD and POD were measured using the SOD and POD Assay Kits (Nanjing JianCheng Bioengineering Institute, Nanjing, China), respectively, according to the manufacturer’s instructions.

### Matchmaker Gold yeast two-hybrid system

The Matchmaker Gold yeast two-hybrid system was used to detect the interactions between AccMKK6, Accp38b, and Acckayak, as described in a previous study [33]. The typical MAPK member *Accp38b* (GenBank accession no. GU321334) was isolated as previously described [34] and ligated into the pGBKT7 vector. The ORFs of *AccMKK6* and *Acckayak* were cloned and fused to the pGADT7 vector. The plasmids Accp38b-pGBKT7 and AccMKK6-pGADT7, Accp38b-pGBKT7, and Acckayak-pGADT7 were co-transformed into the Y2H Gold yeast strain, respectively, and plated on DDO (SD/-Leu/-Trp) medium. Then, the positive clones were grown on selective SD (QDO/X, SD/-Ade/-His/-Leu/-Trp with X-α-gal and Aureobasidin A) medium.

### Glutathione S-transferase pull-down experiments

The ORF of *Accp38b* was ligated into the pGEX-4T-1 vector with the glutathione S-transferase (GST)-tag. The ORF of *AccAP2m* and *AccMKK6* was ligated into the expression vector pET-30a(+) with a His-tag. The recombinant plasmids were then transformed into *E. coli* Transetta (DE3). The pull-down assay was performed using Glutathione sepharose^TM^ 4B (GE Healthcare Life Sciences, Sweden) and eluted with elution buffer. The proteins samples were separated by 12% SDS-PAGE, and the separated proteins were blotted onto PVDF membrane. Western blot analyses were performed using His or GST antibodies (Abmart, Shanghai, China).

## Results

### Bioinformatics and phylogenetic analysis of *AccMKK6*

**Figure 1:**
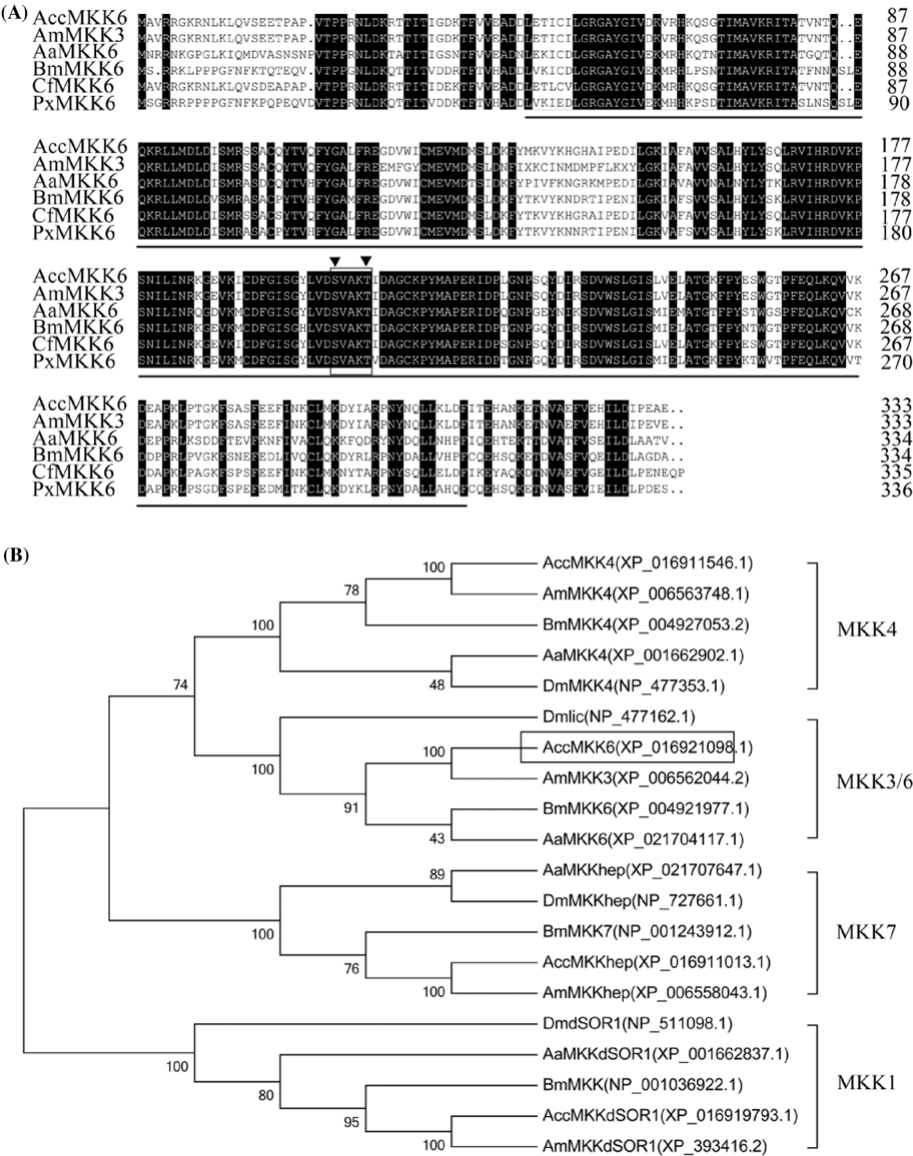
Sequence analysis of *AccMKK6* (**A**) Multiple amino acid sequence alignments for AccMKK6 (*A. cerana cerana*, XP_016921098.1), AmMKK3 (*Apis mellifera*, XP_006562044.2), AaMKK6 (*Aedes aegypti*, XP_021704117.1), BmMKK6 (*Bombyx mori*, XP_004921977.1), CfMKK6 (*Camponotus floridanus*, XP_011266938.1), and PxMKK6 (*Plutella xylostella*, XP_011548047.1). The conserved SxxxT motif is boxed. The S-TKc domains of MKK6 are marked by horizontal lines. The Ser^202^ and Thr^206^ sites are marked by black triangles. (**B**) Phylogenetic analysis of the homologous MKK6 sequences from various species using the neighbor-joining (NJ) method with bootstrap values of 1000 replicates. Four main clades of the MKK superfamily are shown, and AccMKK6 is boxed. The sequences were obtained from the NCBI database.

MKK6 is a novel redox-regulated Rac-binding protein that mediates MAPK cascades and plays important roles in ROS production and disease resistance in many animals [35]. To determine the functions of MKK6 in *A. cerana cerana*, the ORF of *AccMKK6* was obtained by RT-PCR using the specific primers M1F and M1R. The *AccMKK6* ORF consists of 1002 bp and encodes a 333 amino acid protein with a predicted average mass of 37.63 kDa and a theoretical isoelectric point (pI) of 7.15. Multiple sequence analysis of several MKK6s from different species revealed that the amino acid sequence of AccMKK6 has strong similarity to those of AmMKK6 (93.7%), CfMKK6 (91%), PxMKK6 (73.5%), BmMKK6 (73.1%), and AaMKK6 (70.7%). As shown in Figure 1A, AccMKK6 contains typical features of MKKs, including a conserved SxxxT motif and serine/threonine protein kinase catalytic (S-TKc) domain. Based on the amino acid sequences of MKKs from *A. cerana cerana* and other species, an NJ phylogenetic tree was constructed to identify the position of AccMKK6 in the evolutionary history of MKK proteins (Figure 1B). The results showed that AccMKK6 lies within the distinct co-orthologous pattern of insect MKK6s and has a close evolutionary relationship with AmMKK3.

### Developmental stage-specific and tissue-specific expression patterns of *AccMKK6*

**Figure 2:**
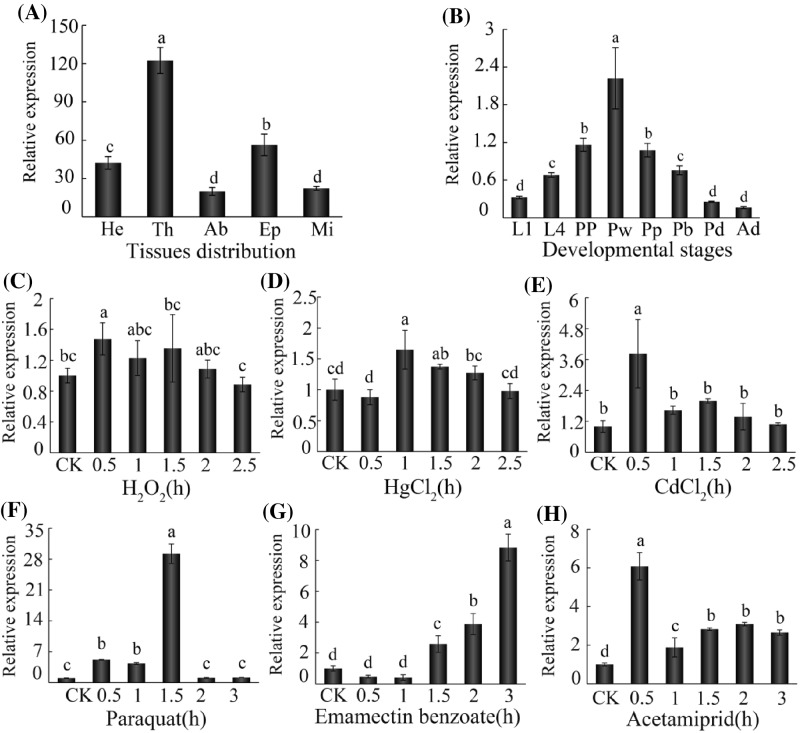
The expression profile of *AccMKK6* determined using qPCR (**A**) Tissue distributions of *AccMKK6* expression: Head (He), thorax (Th), abdomen (Ab), epidermis (Ep), and Midgut (Mi). The tissues were collected from adult worker honeybees. (**B**) The relative expression of *AccMKK6* in different developmental stages: larvae (L1, L4), pupae (PP, Pw, Pp, Pb, and Pd), and adult workers (Ad). (**C**–**H**) Expression profiles of *AccMKK6* under environmental stress conditions. These conditions included (C) H_2_O_2_, (**D**) HgCl_2_, (**E**) CdCl_2_, (**F**) paraquat, (**G**) emamectin benzoate, and (**H**) acetamiprid. Treated 0 h adult worker bees were used as controls. The data are given as the mean ± SE of three replicates. The bars with different letters represent data that are significantly different from each other (*P*<0.05) based on one-way ANOVA and Duncan’s multiple range tests using SPSS software version 17.0.

To analyze the characteristics of *AccMKK6*, we used qPCR to detect the expression patterns of *AccMKK6* in various tissues and in different developmental stages of honeybees. The total RNA was collected from the head, thorax, abdomen, epidermis and midgut. As shown in Figure 2A, *AccMKK6* was expressed in all the tissues that we isolated. Among the tissues examined, the most well-defined expression was observed in the thorax, followed by the epidermis and head. We also analyzed *AccMKK6* expression during different developmental stages and found that *AccMKK6* was highly expressed in the pupal stages. As shown in Figure 2B, *AccMKK6* was highest in the Pw phase. During the larvae stage, the expression levels in L4 were higher than in L1. The expression of *AccMKK6* was lower in the adult stages than in the earlier stages.

### Expression profiles of *AccMKK6* under a variety of environmental stresses

Honeybees are inevitably affected by various external stimuli, including climate change, ecological deterioration, and pathogenic microbial invasion. Therefore, we simulated several abiotic stimuli (H_2_O_2_, heavy metals, and pesticides) to investigate the expression profile of *AccMKK6*, as examined by qPCR. Surprisingly, the relative expression of *AccMKK6* was up-regulated in all of the tested treatments. As shown in Figure 2C, the expression of *AccMKK6* increased with H_2_O_2_ treatment. HgCl_2_ also up-regulated *AccMKK6* expression by 1.6-fold at 1 h, and CdCl_2_ up-regulated *AccMKK6* expression by 3.8-fold at 0.5 h, after which expression returned to the basal level (Figure 2D,E). Honeybee poisoning by pesticides is a serious problem in beekeeping. As shown in Figure 2F–H, *AccMKK6* responded robustly to treatment with a variety of pesticides. The expression of *AccMKK6* increased by 29-fold after 1.5 h of exposure to paraquat. Under exposure to emamectin benzoate, the relative expression level of *AccMKK6* increased by 8.8-fold after 3 h. Moreover, the expression of *AccMKK6* increased more drastically after exposure to acetamiprid. All of these conditions are believed to induce the formation of ROS. These data confirmed that *AccMKK6* may play an important role in the resistance of honeybees to oxidative stress.

### Protein expression levels of AccMKK6 under environmental stresses

**Figure 3:**
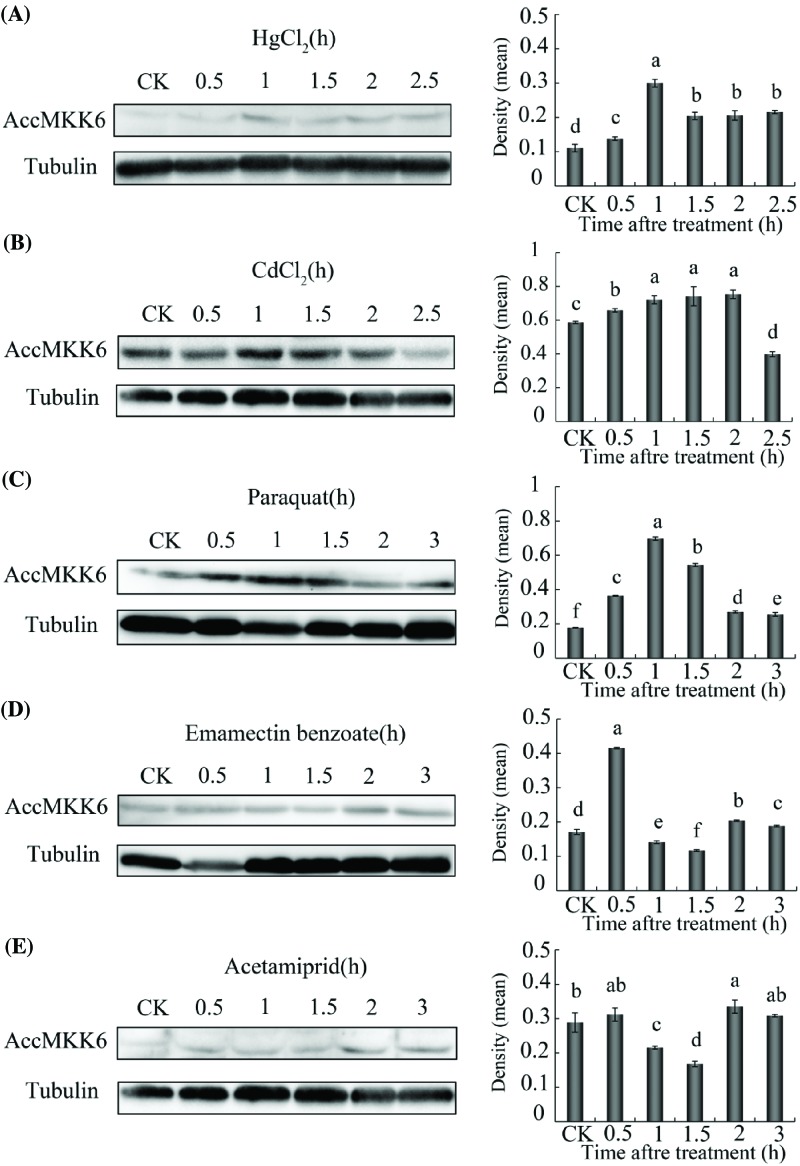
Western blot analysis of *AccMKK6* translation expression levels Honeybees were subjected to the following treatments: (**A**) CdCl_2_, (**B**) HgCl_2_, (**C**) paraquat, (**D**) emamectin benzoate, and (**E**) acetamiprid. *AccMKK6* protein was immunoblotted with anti-AccMKK6. The signal for the binding reaction was visualized with HRP substrate.

To further explore the expression pattern of AccMKK6 in response to various types of oxidative damage, Western blot analysis was used to assess the changes in AccMKK6 after exposure to adverse stress. AccMKK6 was detected using anti-AccMKK6. Density analysis showed that increased AccMKK6 expression was induced after HgCl_2_ treatment at 0.5 h (Figure 3A). As shown in Figure 3B, CdCl_2_ treatment also increased AccMKK6 protein expression levels. Treatment with paraquat and emamectin benzoate up-regulated AccMKK6, and the peak levels were observed at 1 and 0.5 h, respectively (Figure 3C,D). The protein expression of AccMKK6 was reduced after acetamiprid treatment (Figure 3E).

### Effects of *AccMKK6* knockdown on oxidative stress

**Figure 4:**
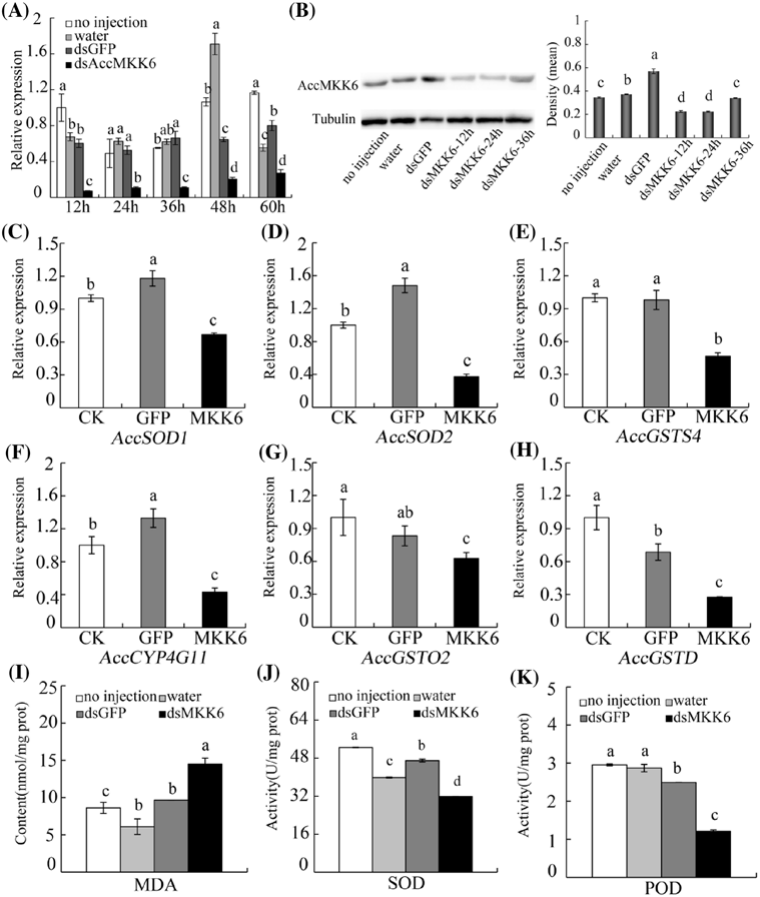
The effects of the RNAi-mediated silencing of *AccMKK6* (**A**) Effects of RNA interference on the mRNA levels of honeybees. (**B**) The expression levels of AccMKK6 protein in control and AccMKK6-silenced honeybees, as measured by Western blot. (**C**–**H**) Expression patterns of other antioxidant genes (*AccSOD1, AccSOD2, AccGSTS4, AccCYP4G11, AccGSTO2*, and *AccGSTD*) at 36 h after *AccMKK6* knockdown, as analyzed by qPCR. (**I**–**K**) Detection of the (I) MDA content and the enzymatic activity of (**J**) SOD and (**K**) POD 36 h after *AccMKK6* knockdown. The data are presented as the means ± SE of three replicates. The bars with different letters represent data that are significantly different from each other (*P*<0.05) based on one-way ANOVA and Duncan’s multiple range tests using SPSS software version 17.0.

RNAi mediated by dsRNA has been used to study gene function in recent years. Here, adult workers were injected with dsAccMKK6-RNA, dsGFP-RNA, or water. The expression of *AccMKK6* was detected by qPCR and Western blot to determine the optimal silencing time. The transcription level of *AccMKK6* in the dsAccMKK6-RNA injection group was significantly inhibited compared with those of the control groups, especially at 12, 24, and 36 h (Figure 4A). In the control groups, the level of *AccMKK6* mRNA fluctuated and then stabilized at 36 h after injection. To clarify the effect of AccMKK6 silencing at the protein level, we performed a Western blot. As shown in Figure 4B, the AccMKK6 expression in the dsAccMKK6-RNA injection group was lower than in the control groups. The above results indicate that dsRNA-mediated gene silencing is successful, and honeybees at 36 h after the dsRNA injection were selected for further study.

To further explore the role of *AccMKK6* in combating oxidative damage, we analyzed the transcription levels of some antioxidant genes at 36 h after *AccMKK6* silencing. The qPCR results in Figure 4C–H showed that *AccSOD1, AccSOD2, AccGSTS4, AccCYP4G11, AccGSTO2*, and *AccGSTD* were down-regulated when *AccMKK6* was knocked down. To confirm the effect of RNAi with *AccMKK6* on the antioxidant capacity of *A. cerana cerana*, we measured the enzymatic activities of SOD and POD and the levels of MDA 36 h after RNAi treatment. The results proved that the MDA concentration in the honeybees in which *AccMKK6* was knocked down were higher than those in the control honeybees (Figure 4I). Moreover, the activities of SOD and POD were lower in the silenced samples than in the control groups (Figure 4J,K).

### AccMKK6 interacted with Accp38b *in vitro*

**Figure 5:**
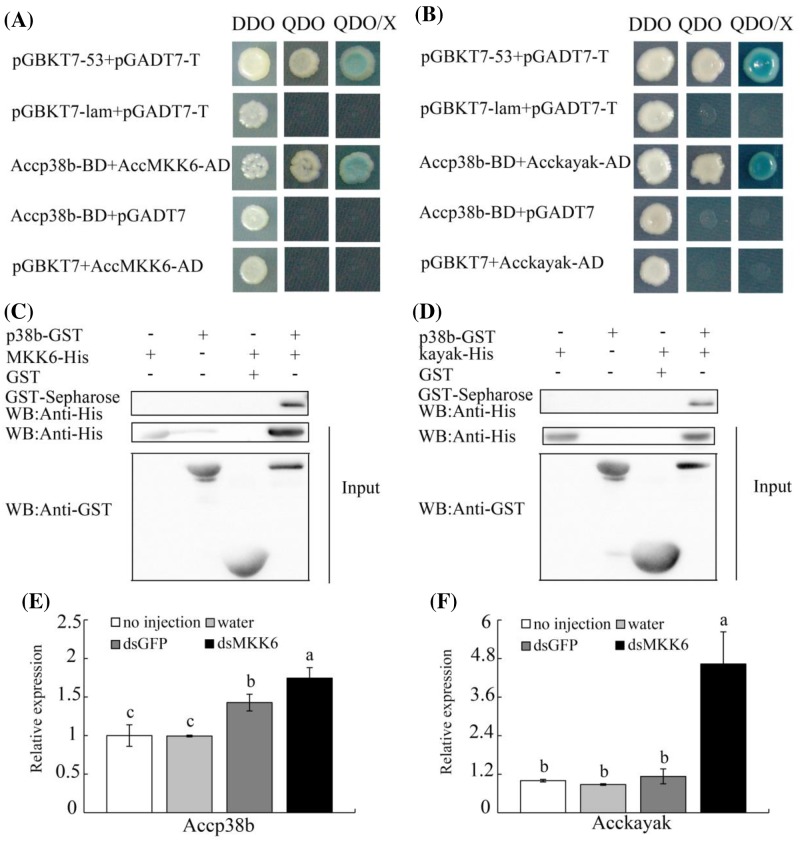
The interaction of AccMKK6, Accp38b, and Acckayak, showing the expression levels of *Accp38b* and *Acckayak* after AccMKK6 silencing (**A** and **B**) The fusion construct pair AccMKK6-AD and Accp38b-BD and the pair Acckayak-AD and Accp38b-BD fusion constructs were co-transformed into the Y2H Gold yeast strain and grown on DDO and QDO SD media. The positive clones were confirmed on QDO/X SD media. (**C** and **D**) The binding of AccMKK6 and Accp38b and of Acckayak and Accp38b protein were analyzed by GST pull-down experiments. (**E** and **F**) The expression profiles of *Acckayak* and *Accp38* after *AccMKK6* knockdown.

Previous studies have reported that MKK6 mediates the activation of p38 MAPK in the response to oxidative stress [36]. To investigate the downstream component of *AccMKK6*, based on our previous research and the honeybee genome, we detected the interactions between AccMKK6 and Accp38b using yeast two-hybrid assays. The yeast two-hybrid assay results showed that the positive control clone and the clone co-transformed with AccMKK6 and Accp38b grew well on SD medium DDO and QDO plates (Figure 5A). To verify the yeast two-hybrid results, we performed GST pull-down assays. As shown in Figure 5C, the AccMKK6 protein was captured and specifically bound to p38b protein, according to an assay using the antibody against the His epitope. These results indicated that AccMKK6 interacts with Accp38b.

### Cloning and characterization of *Acckayak*

**Figure 6:**
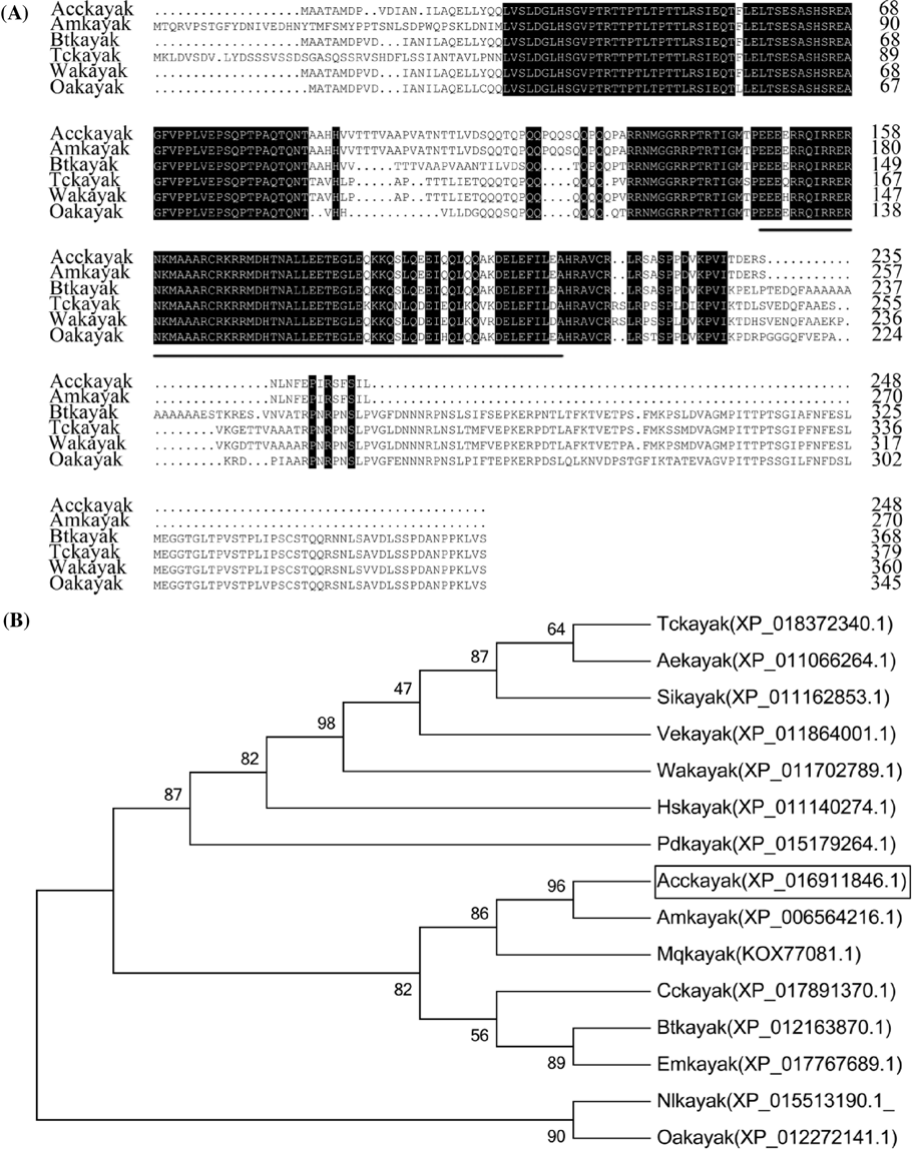
Sequence analysis of *Acckayak* (**A**) Multiple amino acid sequence alignments for Acckayak (*Apis cerana cerana*, XP_016911846.1), Amkayak (*Apis mellifera*, XP_006564216.1), Wakayak (*Wasmannia auropunctata*, XP_011702789.1), TcMKK6 (*Trachymyrmex cornetzi*, XP_018372340.1), Btkayak (*Bombus terrestris*, XP_012163870.1), and OaMKK6 (*Orussus abietinus*, XP_012272141.1). The bZIP domain of kayak is marked by horizontal lines. (**B**) Phylogenetic analysis of the homologous kayak sequences from various species obtained using the neighbor-joining (NJ) method with bootstrap values of 1000 replicates. Acckayak is boxed. The sequences were obtained from the NCBI database.

The transcription factor kayak is among the most important substrates for MAPK signal pathways [37]. Therefore, kayak was cloned to investigate the involvement of *AccMKK6* in the MAPK cascades in *A. cerana cerana*. The ORF of *Acckayak* is 747 bp in length and encodes a putative polypeptide of 248 amino acid residues with a predicted molecular mass of 27.73 kDa and a theoretical pI of 6.8. Multiple sequence analysis of several kayaks from different species revealed that the deduced amino acid sequence of Acckayak is highly homologous to those of Amkayak, Btkayak, Oakayak, Tckayak, and Wakayak. Acckayak shared an average similarity of 70.59% with kayak sequences from other eukaryotic organisms, which suggested that kayak is highly conserved across species. As shown in Figure 6A, Acckayak contained a conserved basic leucine zipper protein (bZIP) domain. An NJ phylogenetic tree was constructed to understand the evolutionary relationships among kayaks (Figure 6B). Phylogenetic analysis revealed that the homologous protein Amkayak shares the highest similarity with Acckayak.

### Accp38b interacted with Acckayak, and both were induced by *AccMKK6* silencing

To investigate the interaction between Accp38 and Acckayak, a yeast two-hybrid assay was performed. As shown in Figure 5B, Accp38b and Acckayak were co-transformed into the Y2H Gold yeast strain, which grew well on the QDO plates. The interaction between Accp38b and Acckayak was further confirmed using a GST pull-down assay. As shown in Figure 5D, the Acckayak protein was captured and specifically bound to p38b protein, according to the assay using the antibody against the His epitope. These results indicated that Accp38b interacts with Acckayak. In addition, the expression levels of *Acckayak* and *Accp38b* were examined after *AccMKK6* silencing. Both *Acckayak* and *Accp38b* had higher expression levels in AccMKK6-silenced honeybees than in control honeybees (Figure 5E,F).

### Expression profiles of *Acckayak* under a variety of environmental stresses

**Figure 7:**
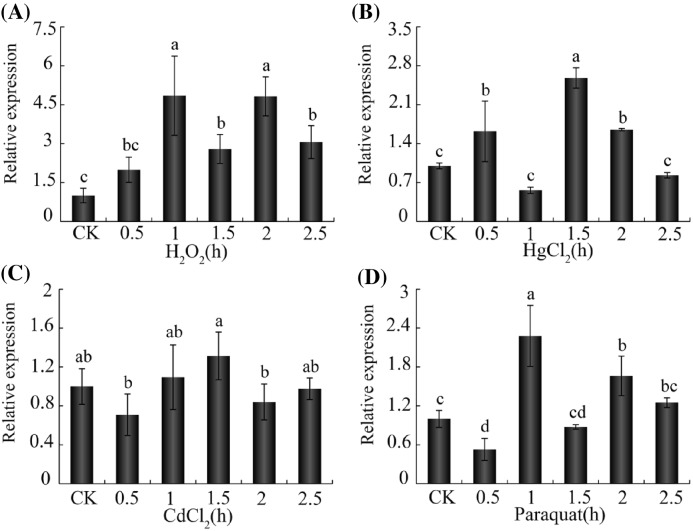
The expression profiles of *Acckayak* determined using qPCR Expression profiles of *Acckayak* under environmental stress. These conditions included (**A**) H_2_O_2_, (**B**) HgCl_2_, (**C**) CdCl_2_, and (**D**) paraquat. Treated 0 h adult worker bees were used as controls. The data are given as the mean ± SE of three replicates. The bars with different letters represent data that are significantly different from each other (*P*<0.05) based on one-way ANOVA and Duncan’s multiple range tests using SPSS software version 17.0.

To explore the relationship between AccMKK6 and Acckayak, we analyzed the expression patterns of *Acckayak* after several stress treatments. As shown in Figure 7, the expression of *Acckayak* was induced after the H_2_O_2_, HgCl_2_, CdCl_2_, and paraquat treatments. Notably, the expression patterns of *Acckayak* were approximately consistent with those of *AccMKK6* after the same treatments.

## Discussion

The MAPK signaling pathway is one of the important signal transduction systems in organisms, and many MKKs have been identified. MKKs are a family of serine/threonine protein kinases that are involved in many cellular responses including cell growth, nervous system development and immunity [17,38]. Previous studies demonstrated that MKKs may play a role in combatting oxidative stress [12]. Thus, we guessed that *AccMKK6* might participate in the oxidative stress response and have conducted a series of experiments to test our prediction.

MKK6 is a member of the dual-specificity protein kinase family and is activated upon the phosphorylation of serine and threonine residues by upstream MKKKs [39]. The serine/threonine protein kinase catalytic domain (S-TKc) was found in the polypeptide sequence of AccMKK6 subject to protein phosphorylation (residues 48–309), and Ser^202^ and Thr^206^ sites were identified. Sequence analysis revealed that AccMKK6 contained sequences that are highly conserved among other typical MKK6s in insects. As shown in the phylogenetic tree, different MKKs from insects were clearly clustered into four clades, and AccMKK6 belongs to the MKK3/6 clade, sharing the highest degree of homology with the MKK3 protein from *Apis mellifera*.

Understanding the stage and tissue distributions of *AccMKK6* mRNA may help to better understand its functional mechanisms. Therefore, we analyzed *AccMKK6* mRNA expression at multiple developmental stages and examined its tissue distribution. The transcription level of *AccMKK6* was strikingly higher in the pupal stages than in the other stages. Because ROS accumulation can cause oxidative damage, particularly in fast-growing organisms [40], we hypothesized that *AccMKK6* is essential for honeybee development and may prevent ROS-mediated oxidative damage during the pupae growth stages. Tissue-specific expression analysis showed that *AccMKK6* is expressed in various tissues but is most highly expressed in the thorax and epidermis. Similarly, the highest expression of *Accp38b* has been detected in the thorax [34]. The epidermis acts as the exoskeleton, conferring physical stability, and participates in the responses to various environmental stresses [41]. These data indicated that *AccMKK6* might be involved in protecting honeybees against harm from oxidative stress.

Honeybees suffer constantly from a variety of adverse environmental stresses, and their number has plummeted [42]. H_2_O_2_ is a typical oxidant that induces ROS elevation and cell death [43]. Heavy metal ions play essential roles in many physiological processes, and excess heavy metals can activate the MAPK cascades to interfere with cellular oxygen metabolism [44]. Moreover, Cd^2+^ and Hg^2+^ exposure enhances lipid peroxidation and ROS generation and alters the level of antioxidant enzymes [45,46]. Pesticides are significant threats to a honeybee’s life and can leading to impaired biochemical and physiological functions [47]. For instance, emamectin benzoate is a novel macrocyclic lactone insecticide that can induce intracellular ROS accumulation and cell apoptosis [48]. Paraquat is one of the most widely used herbicides in agriculture and is a strong redox agent that yields paraquat monocation radicals, which then react with molecular oxygen to produce superoxide anions [49]. In *A. mellifera*, the neonicotinoid insecticide acetamiprid acts on acetylcholine nicotinic receptors and affects behavior [50]. After the exposure of *Cydia pomonella* to acetamiprid, the activity of CYP450 activity increased, suggesting activation of the antioxidant system to maintain redox homeostasis [51]. Therefore, we simulated several adverse life-threatening environmental conditions that could cause ROS damage in *A. cerana cerana* and explored the resulting changes in *AccMKK6* expression at the transcription levels to look for evidence of their antioxidant functionality under environmental stress. We found that the expression level of *AccMKK6* was up-regulated after H_2_O_2_, CdCl_2_ and HgCl_2_ treatment. Our results also revealed that the pesticide treatments affected the expression profiles of *AccMKK6*. As we all know, the gene expression pattern is usually an indicator of its function. Studies have shown that organisms repair oxidative damage by altering the expression of related genes, including by up-regulating antioxidant genes [52]. These results collectively indicated that *AccMKK6* might protect honeybees from ROS-induced damage under oxidative stress.

We also detected the expression of AccMKK6 at the protein level after HgCl_2_, CdCl_2_, emamectin benzoate, and acetamiprid treatments. The results were not completely consistent with the mRNA expression data. The protein-to-mRNA ratio is different for different genes; additionally, the ratio might change after different treatments [53]. The stability of the transcript itself and the efficiency of translation and the stability of the translated product will affect the expression of the final product, as will problems with post-translational processing.

The RNA-mediated inhibition of endogenous target gene expression has become a popular strategy for determining gene function. An RNAi experiment was performed to further investigate the function of *AccMKK6. AccMKK6* was successfully knocked down by RNAi. Organisms have many systems to control oxidative damage caused by ROS. Cellular antioxidant systems consist of multifunctional proteins and enzymes [54]. The expression of antioxidant genes changed after *AccMKK6* knockdown, indicating that *AccMKK6* may response to oxidative stress. By investigating the transcription levels of most of the antioxidant genes, such as *AccSOD1, AccSOD2, AccGSTS4, AccCYP4G11, AccGSTO2*, and *AccGSTD*, we found that they were reduced when AccMKK6 was knocked down. Previous studies reported that *AccYB-1* may play an important role in response to oxidative damage [55]. Nrf can restrain the expression of antioxidant enzymes to maintain the activity of cellular defenses and/or to rapidly restore induced enzymes to normal levels [56]. We analyzed the transcription levels of *AccYB-1* and *AccNrf* in *AccMKK6*-silenced honeybees and found that *AccYB-1* and *AccNrf* were induced when *AccMKK6* was silenced (Supplementary Figure S1). These results indicate that the AccMKK6 signal cascade plays a role in oxidative stress.

Lipid oxidation results in the generation of many harmful secondary products, and MDA can act as a reliable indicator of lipid peroxidation, indirectly reflecting cellular damage [57]. When AccMKK6 was silenced in honeybees, lipid peroxides began to accumulate faster, resulting in higher MDA concentrations. SOD and POD, as antioxidant enzymes, are the first line of defense that acts directly on ROS attacks [40]. SODs convert superoxide to oxygen into H_2_O_2_, and then PODs convert the H_2_O_2_ into H_2_O [58]. The activity levels of these enzymes were changed by *AccMKK6* knockdown, which may result in elevated ROS levels. Based on these findings, we conclude that the *AccMKK6* may possess potent antioxidant properties.

Previous studies have shown that in mammals, MKK6 regulates a variety of fundamental cellular processes involved in the response to environmental stimuli by participating in the MAPK cascades [59,60]. In the p38 MAPK cascade, MKK3, MKK4, and MKK6 serve as upstream MAPK kinases responsible for p38 activation, and while downstream targets of p38 are either other kinases or transcription factors such as ATF-2 and MEF2 [14,61]. The qPCR results showed that the transcriptional factors ATF2 and MEF2 were responsive to environmental and chemical stresses (Supplementary Figure S2). These results suggest that MAPK cascade pathways play an important role in the response to various stresses in *A. cerana cerana*. To verify whether *AccMKK6* participates in the MAPK cascades in *A. cerana cerana*, we cloned the transcription factor kayak from *A. cerana cerana* and named it *Acckayak*. Yeast two-hybrid analysis indicated that AccMKK6 interacts with Accp38b, and Accp38b interacts with Acckayak. At the same time, we also found that the expression levels of *Accp38b* and *Acckayak* were up-regulated after *AccMKK6* was silenced, which supports the participation of *AccMKK6* in the MAPK cascades in *A. cerana cerana*. A recent study reported CAR binding and inhibition of the GADD45B-MKK6 scaffold to repress the phosphorylation of p38 MAPK [62]. The *Drosophila fos (Dfos)/kayak* gene is required in follicle cells for the dumping of the nurse cell cytoplasm into the oocyte and the subsequent apoptosis of nurse cells [37]. After the silencing of *AccMKK6*, the antioxidant capacity of bees decreased and the expression of *Accp38b* and *Acckayak* was up-regulated. Therefore, we hypothesized that *AccMKK6* can enhance the antioxidant capacity of bees by inhibiting the expression of *Accp38b* and *Acckayak*.

High concentrations of ROS will lead to a various types of oxidative damage such as DNA strand breaks, biofilm lipid peroxidation and protein oxidative degeneration, which eventually lead to accelerated cell death or apoptosis and may even cause diseases [1,63]. Therefore, understanding the antioxidant system and its mechanism of defense against ROS is critical for the survival of organisms. Here, we have revealed via bioinformatics, phylogenetic analysis, expression analysis and RNAi that *AccMKK6* exists in honeybees and play an important role in the mechanism of the oxidative stress response. Meanwhile, we clarified the relationship between *AccMKK6, Accp38b*, and *Acckayak* through the interaction study and described the involvement of *AccMKK6* in the MAPK cascades, laying a foundation for the study of the MAPK cascades in *A. cerana cerana*.

## Supporting information

**Supplemental Figure 1:** The expression profile of (A) Y-box and (B) Nrf after AccMKK6 konckdown, as measured by qPCR. The data are given as the mean ± SE of three replicates. The bars with different letters represent data that are significantly different from each other (P < 0.05) based on one-way ANOVA and Duncan's multiple range tests using SPSS software version 17.0.

**Supplemental Figure 2:** The expression profile of ATF2 and MEF2 determined using qPCR. (A-C) Expression profiles of ATF2 under environmental stress conditions. These conditions included (A) H2O2, (B) HgCl2 and (C) acetamiprid. (D-F) Expression profiles of MEF2 under environmental stress conditions. These conditions included (D) H2O2, (E) HgCl2 and (F) acetamiprid. The data are given as the mean ± SE of three replicates. The bars with different letters represent data that are significantly different from each other (P < 0.05) based on one-way ANOVA and Duncan's multiple range tests using SPSS software version 17.0.

**Supplemental Table 1:** Primer sequences used in this research.

**Supplemental Table 2:** Procedures used in this study.

## Abbreviations

bZIP: basic leucine zipper protein
ER: endoplasmic reticulum
GST: glutathione S-transferase
MAPK: mitogen-activated protein kinase
MDA: malonyldialdehyde
MKK: mitogen-activated protein kinase kinase
NJ: neighbor-joining
ORF: open reading frame
POD: peroxidase
PVDF: polyvinylidene difluoride
RNAi: RNA interference
ROS: reactive oxygen species
SDS-PAGE: sodium dodecyl sulfate polyacrylamide gel electrophoresis
SOD: superoxide dismutase
