# Supplementary material for: Molecular mechanism by which Apis cerana cerana MKK6 (AccMKK6)-mediated MAPK cascades regulate the oxidative stress response
Source: Biosci Rep. 2018 Dec 11;38(6):BSR20181301. doi: 10.1042/BSR20181301 (PMC6294647; doi:10.1042/BSR20181301)
Supplement: Supplementary file 1 [file bsr20181301_Supp1.pdf]

Supplemental Figure 1

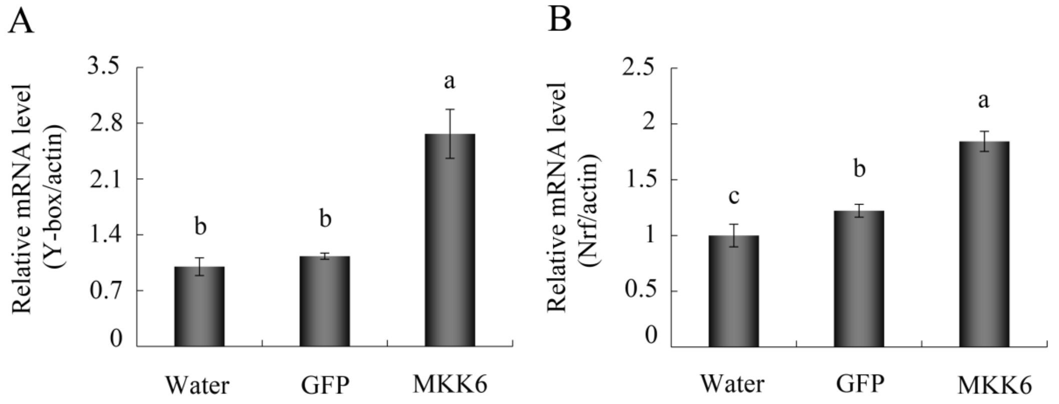

Supplemental Figure 1: The expression profile of (A) Y-box and (B) Nrf after AccMKK6 konckdown, as measured by qPCR. The data are given as the mean ± SE of three replicates. The bars with different letters represent data that are significantly different from each other ($P < 0.05$) based on one-way ANOVA and Duncan's multiple range tests using SPSS software version 17.0.

Supplemental Figure 2

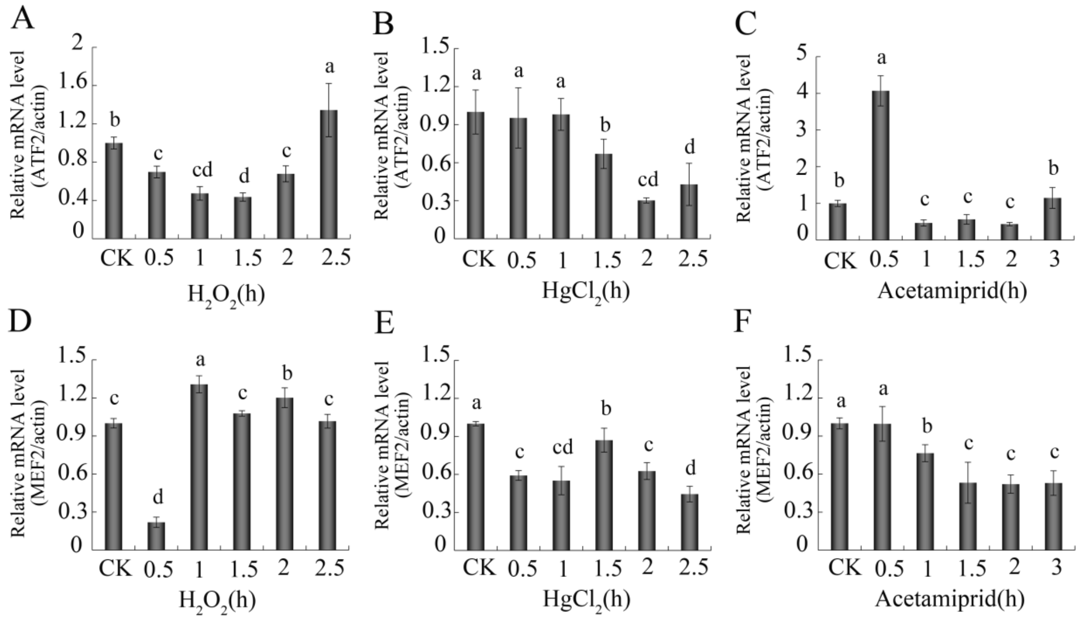

Supplemental Figure 2: The expression profile of ATF2 and MEF2 determined using qPCR. (A-C) Expression profiles of ATF2 under environmental stress conditions. These conditions included (A) H_2O_2, (B) $HgCl_2$ and (C) acetamiprid. (D-F) Expression profiles of MEF2 under environmental stress conditions. These conditions included (D) H_2O_2, (E) $HgCl_2$ and (F) acetamiprid. The data are given as the mean ± SE of three replicates. The bars with different

letters represent data that are significantly different from each other ($P < 0.05$) based on one-way ANOVA and Duncan's multiple range tests using SPSS software version 17.0.

Supplemental Table1. Primer sequences used in this research.

**Abbreviation**	**Primer sequence (5'-3')**	**Description**
M1F	GGCTGTACGTCGTGGAAAAAG	cDNA sequence primer of *AccMKK6*, forward
M1R	TTCAGCTTCAGGAATATCTAAAATATGTTCAAC	cDNA sequence primer of *AccMKK6*, reverse
K1F	CGCCATGGATCCAGTCGAC	cDNA sequence primer of *Acckayak*, forward
K1R	CACAGTATTGAAAAACTTCGAATCGG	cDNA sequence primer of *Acckayak*, reverse
P1F	GCCGCAGTTTCATAAAGTCG	cDNA sequence primer of *Accp38*, forward
P1R	CGAAGTAGACTCAATACCCG	cDNA sequence primer of *Accp38*, reverse
M2F	GGATCCATGGCTGTACGTCGTGGAAAAAG	Protein expression primer of *AccMKK6* , forward
M2R	GAGCTCAATTCAGCTTCAGGAATATCTAAAATATGTTCAAC	Protein expression primer of *AccMKK6* , reverse
M3F	TAATACGACTCACTATAGGGCGACTTGTAGATTCTGTTGCCAAA	RNAi primer of *AccMKK6*, forward
M3R	TAATACGACTCACTATAGGGCGAGCAACATTTGTTTCCTTATTG	RNAi primer of *AccMKK6*, reverse
M4F	GAATTC ATGGCTGTACGTCGTGG	pGADT7 construction primer of *AccMKK6*, forward
M4R	GGATCCTTCAGCTTCAGGAATATCTAAAATATG	pGADT7 construction primer of *AccMKK6*, reverse
K2F	CATATG ATGGCAGCCACCGCCATG	pGADT7 construction primer of *Acckayak*, forward
K2R	GAATTCCAGTATTGAAAAACTTCGAATCGGTTC	pGADT7 construction primer, of *Acckayak* reverse
P2F	GAATTCATGCCGCAGTTTCATAAAGTCG	pGBKT7 construction primer of *Accp38b*, forward

P2R	GGATCCCGAAGTAGACTCAATACCCG	pGBKT7 construction primer *Accp38b* , reverse
β-s	TTATATGCCAACACTGTCCTTT	Standard control primer, forward
β-x	AGAATTGATCCACCAATCCA	Standard control primer, reverse
M5F	GGAGAAGTTAAGATTTGTGACTTTGG	qPCR primer of *AccMKK6*, forward
M5R	CCAGTTGGTAATTTGGGTGCTTC	qPCR primer of *AccMKK6*, reverse
GSTS4F	CTTCTTAGTTATGGAGGTGTTG	qPCR primer of *AccGSTS4*, forward
GSTS4R	GCCATCTGAAATCGTAAAGAG	qPCR primer of *AccGSTS4*, reverse
SOD1F	AAACTATTCAACTTCAAGGACC	qPCR primer of *AccSOD1*, forward
SOD1R	CACAAGCAAGACGAGCACC	qPCR primer of *AccSOD1*, reverse
SOD2F	TTGCCATTCAAGGTTCTGGTT	qPCR primer of *AccSOD2*, forward
SOD2R	GCATGTTCCCAAACATCAATACC	qPCR primer of *AccSOD2*, reverse
GSTO2F	CCAGAAGTAAAAGGACAAGTTCGT	qPCR primer of *AccGSTO2*, forward
GSTO2R	CCATTAACATCAACAAGTGCTGGT	qPCR primer of *AccGSTO2*, reverse
GSTDF	CGAAGGAGAAAACTATGTGGCAG	qPCR primer of *AccGSTD*, forward
GSTDR	CGTAATCCACCACCTCTATCG	qPCR primer of *AccGSTD*, reverse
CYP4G11F	CGCAAAGAGAATGGGAAGG	qPCR primer of *AccCYP4G11*, forward
CYP4G11R	CTTTTGTGTACGGAGGTGC	qPCR primer of *AccCYP4G11*, reverse

Supplemental Table2. Procedures used in this study.

Primers pair	Amplification conditions
M1F/M1R	10 min at 94 °C, 40 s at 94 °C, 40 s at 52 °C, 1 min at 72 °C for 35 cycles, 10 min at 72 °C
M2F/M2R	10 min at 94 °C, 40 s at 94 °C, 40 s at 57 °C, 1 min at 72 °C for 35 cycles, 10 min at 72 °C
M3F/M3R	10 min at 94 °C, 40 s at 94 °C, 40 s at 60°C, 30 s at 72 °C for 35 cycles, 10 min at 72 °C
M4F/M4R	10 min at 94 °C, 40 s at 94 °C, 40 s at 54 °C, 1 min at 72 °C for 35 cycles, 10 min at 72 °C
K1F/K1R	10 min at 94 °C, 40 s at 94 °C, 40 s at 53 °C, 1 min at 72 °C for 35 cycles, 10 min at 72 °C
K2F/K2R	10 min at 94 °C, 40 s at 94 °C, 40 s at 56 °C, 1 min at 72 °C for 35 cycles, 10 min at 72 °C
P1F/P1R	10 min at 94 °C, 40 s at 94 °C, 40 s at 50 °C, 1 min at 72 °C for 35 cycles, 10 min at 72 °C
P2F/P2R	10 min at 94 °C, 40 s at 94 °C, 40 s at 55 °C, 1 min at 72 °C for 35 cycles, 10 min at 72 °C
